# Supplementary material for: Gram Stain and Culture of Sputum Samples Detect Only Few Pathogens in Community-Acquired Lower Respiratory Tract Infections: Secondary Analysis of a Randomized Controlled Trial
Source: Diagnostics (Basel). 2023 Feb 8;13(4):628. doi: 10.3390/diagnostics13040628 (PMC9955084; doi:10.3390/diagnostics13040628)
Supplement: Supplementary file 1 [file diagnostics-13-00628-s001.zip › diagnostics-2120038-supplementary.pdf]

Supplementary material

Gram stain and culture of sputum samples are of limited diagnostic value: Secondary analysis of a randomized controlled trial

Mariana B. Cartuliares, Helene Skjøt-Arkil, Christian B. Mogensen, Thor A. Skovsted, Steen L. Andersen, Andreas K. Pedersen, and Flemming S. Rosenvinge

Supplementary Table S1: Sensitivity analysis of Gram stain and culture from samples obtained by TS (TS-SG, TS-IG) and FET.

Findings		Sampling method		Total	p-value
		TS	FET		
Total (%)		177 (78%)	50 (22%)	227	
**Gram stain**					
	Number of positive samples	31 (18%)	15 (30%)	46 (20%)*	
	All potential pathogens	39 (22%)	22 (44%)	61 (27%)*	0.851
	Gram-positive cocci chains/pairs	15 (38%)	6 (27%)	21 (34%)#	
	Gram-positive cocci clusters	3 (8%)	2 (9%)	5 (8%)#	
	Gram-negative rods	5 (13%)	6 (27%)	11 (18%)#	
	Gram-positive rods	2 (5%)	1 (5%)	3 (5%)#	
	Gram-positive single	5 (13%)	3 (14%)	8 (13%)#	
	Gram-negative diplococci	3 (8%)	2 (9%)	5 (8%)#	
	Yeast	6 (15%)	2 (9%)	8 (13%)#	
	Upper airway microbiota	57 (32%)	28 (56%)	85 (37%)*	0.009
**Culture**					
	Number of positive samples	63 (36%)	18 (36%)	81 (36%)*	
	All potential pathogens	72 (41%)	21 (42%)	93 (41%)*	0.682
	Streptococcus pneumoniae	4 (6%)	1 (5%)	5 (5%)#	
	Enterococcus sp.	2 (3%)	0 (0%)	2 (2%)#	
	Staphylococcus aureus	19 (26%)	2 (10%)	21 (23%)#	
	Haemophilus influenzae	3 (4%)	2 (10%)	5 (5%)#	
	Enterobacterales	25 (35%)	10 (48%)	35 (38%)#	
	Moraxella catarrhalis	3 (4%)	2 (10%)	5 (5%)#	
	Pseudomonas aeruginosa	1 (1%)	0 (0%)	1 (1%)#	
	Other	1 (1%)	0 (0%)	1 (1%)#	
	Yeast	14 (19%)	4 (19%)	18 (19%)#	
	Upper airway microbiota	27 (15%)	7 (14%)	34 (15%)*	0.836
	No growth of pathogens	67 (38%)	16 (32%)	83 (37%)*	0.519

*Percentage of total #Percentage of all potential pathogens. TS (TS-SG and TS-IG) Tracheal secretion from the standard care group and Tracheal secretion from the intervention group FET: Forced expiratory technique

Supplementary Table S2: Sensitivity analysis of Gram stain and culture from samples obtained by TS (TS-SG, TS-IG) and IS.

Findings		Sampling method		Total	p-value
		TS	IS		
Total (%)		177 (75%)	58 (25%)	235	
**Gram stain**					
	Number of positive samples	31 (18%)	13 (22%)	44 (19%)*	
	All potential pathogens	39 (22%)	14 (24%)	53 (23%)*	0.210
	Gram-positive cocci chains/pairs	15 (38%)	4 (29%)	19 (36%)#	
	Gram-positive cocci clusters	3 (8%)	1 (7%)	4 (8%)#	
	Gram-negative rods	5 (13%)	6 (43%)	11 (21%)#	
	Gram-positive rods	2 (5%)	0 (0%)	2 (4%)#	
	Gram-positive single	5 (13%)	1 (7%)	6 (11%)#	
	Gram-negative diplococci	3 (8%)	2 (14%)	5 (9%)#	
	Yeast	6 (15%)	0 (0%)	6 (11%)#	
	Upper airway microbiota	57 (32%)	32 (55%)	89 (38%)*	0.008
**Culture**					
	Number of positive samples	63 (36%)	24 (41%)	87 (37%)*	
	All potential pathogens	72 (41%)	27 (47%)	99 (42%)*	0.391
	Streptococcus pneumoniae	4 (6%)	1 (4%)	5 (5%)#	
	Enterococcus sp.	2 (3%)	0 (0%)	2 (2%)#	
	Staphylococcus aureus	19 (26%)	2 (7%)	21 (21%)#	
	Haemophilus influenzae	3 (4%)	2 (7%)	5 (5%)#	
	Enterobacterales	25 (35%)	10 (37%)	35 (35%)#	
	Moraxella catarrhalis	3 (4%)	1 (4%)	4 (4%)#	
	Pseudomonas aeruginosa	1 (1%)	1 (4%)	2 (2%)#	
	Other	1 (1%)	2 (7%)	3 (3%)#	
	Yeast	14 (19%)	8 (30%)	22 (22%)#	
	Upper airway microbiota	27 (15%)	8 (14%)	35 (15%)*	0.797
	No growth of pathogens	67 (38%)	15 (26%)	82 (35%)*	0.154

*Percentage of total #Percentage of all potential pathogens. TS (TS-SG and TS-IG) Tracheal secretion from the standard care group and Tracheal secretion from the intervention group. IS: Induced sputum

Supplementary Table S3: Sensitivity analysis of the detected pathogens in relation to antibiotic treatment (within one month before admission) stratified by the collected methods TS and FET, and TS and IS.

Detected pathogens from TS-SG, TS-IG, and IS	**Antibiotics (NO)** n=103 (44%)	**Antibiotic (YES)** n=132 (56%)	**Total** n=235	**p-value**
Common pathogens of CA-LRTI	9 (9%)	5 (4%)	14 (6%)*	0.025
Possible pathogens of CA-LRTI	13 (13%)	10 (8%)	23 (10%)*	0.030
Unlikely pathogens of CA-LRTI	15 (15%)	47 (36%)	62 (26%)*	<0.001
Detected pathogens from TS-SG, TS-IG, and FET	**Antibiotics (NO)** n=102 (45%)	**Antibiotic (YES)** n=125 (55%)	**Total** n=227	**p-value**
Common pathogens of CA-LRTI	10 (10%)	5 (4%)	1 (<1%)*	0.015
Possible pathogens of CA-LRTI	13 (13%)	9 (7%)	22 (10%)*	0.025
Unlikely pathogens of CA-LRTI	13 (13%)	43 (34%)	56 (25%)*	<0.001

*Percentage of total. TS-SG: Tracheal secretion from the standard care group, TS-IG: Tracheal secretion from the intervention group, FET: Forced expiratory technique, IS: Induced sputum.

Supplementary Table S4: Identified microorganisms and upper airway microbiota in samples from patients untreated and treated with antibiotics (within one month before admission).

		**Antibiotic (NO)** n=128 (45%)	**Antibiotic (YES)** n=157 (55%)	**Total** n=285	**p-value**
**Culture**					
	Number of positive samples	40 (31%)	65 (41%)	105 (37%)*	
	All potential pathogens	43 (34%)	77 (49%)	120 (42%)*	0.004
	S.pneumoniae	5 (11%)	1 (1%)	6 (5%)[#]	
	Enterococcus sp.	0 (0%)	2 (3%)	2 (2%)[#]	
	S.aureus	13 (30%)	10 (13%)	23 (19%)[#]	
	H.influenzae	3 (7%)	4 (5%)	7 (6%)[#]	
	Enterobacteriaceae	11 26%)	34 (44%)	45 (37%)[#]	
	M.catarrhalis	4 (9%)	2 (3%)	6 (5%)[#]	
	P.aeruginosa	1 (2%)	1 (1%)	2 (2%)[#]	
	Yeast	4 (9%)	22 (29%)	26 (22%)[#]	
	Other	2 (5%)	1 (1%)	3 (3%)[#]	
	Upper airway microbiota	18 (14%)	24 (15%)	42 (15%)*	0.784
	No pathogen growth	49 (38%)	49 (31%)	98 (34%)*	0.282

*Percentage of total [#]Percentage of all potential pathogens.
